# Antihypertensive medication classes and the risk of dementia over a decade of follow-up

**DOI:** 10.1097/HJH.0000000000003324

**Published:** 2022-11-18

**Authors:** Jakob L. Schroevers, Esmé Eggink, Marieke P. Hoevenaar-Blom, Jan Willem Van Dalen, Tessa Van Middelaar, Willem A. Van Gool, Edo Richard, Eric P. Moll Van Charante

**Affiliations:** a Department of General Practice/Family Medicine; b Department of Public and Occupational Health, Amsterdam University Medical Centre, University of Amsterdam, Amsterdam; c Department of Neurology, Donders Institute for Brain, Behaviour and Cognition, Radboud University Medical Centre, Nijmegen; d Department of Neurology, Amsterdam University Medical Centre, University of Amsterdam, Amsterdam, the Netherlands

**Keywords:** antihypertensives, dementia, hypertension, prevention, primary care

## Abstract

**Introduction:**

Use of angiotensin II (ATII)-stimulating antihypertensive medication (AHM), including angiotensin receptor blockers (ARBs) and dihydropyridine calcium channel blockers (CCBs), has been associated with lower dementia risk. Previous studies had relatively short follow-up periods. The aim of this study is to investigate if these effects are sustained over longer periods.

**Methods:**

This post hoc observational analysis was based on data from a dementia prevention trial (preDIVA and its observational extension), among Dutch community-dwelling older adults without prior diagnosis of dementia. Differential associations between AHM classes and incident dementia were studied after 7.0 and 10.4 years, based on the median follow-up durations of dementia cases and all participants.

**Results:**

After 7 years, use of ATII-stimulating antihypertensives [hazard ratio = 0.68, 95% confidence interval (CI) = 0.47–1.00], ARBs (hazard ratio = 0.54, 95% CI = 0.31–0.94) and dihydropyridine CCBs (hazard ratio = 0.52, 95% CI = 0.30–0.91) was associated with lower dementia risk. After 10.4 years, associations for ATII-stimulating antihypertensives, ARBs and dihydropyridine CCBs attenuated (hazard ratio = 0.80, 95% CI = 0.61–1.04; hazard ratio = 0.75, 95% CI = 0.53–1.07; hazard ratio = 0.73, 95% CI = 0.51–1.04 respectively), but still suggested lower dementia risk when compared with use of other AHM classes. Results could not be explained by competing risk of mortality.

**Conclusion:**

Our results suggest that use of ARBs, dihydropyridine CCBs and ATII-stimulating antihypertensives is associated with lower dementia risk over a decade, although associations attenuate over time. Apart from methodological aspects, differential effects of antihypertensive medication classes on incident dementia may in part be temporary, or decrease with ageing.

## INTRODUCTION

Dementia is a major global health problem, which is expected to increase over the coming years, because of global aging [1]. Results from several prospective studies suggest that hypertension is a risk factor for late-life dementia, in particular vascular dementia and Alzheimer's disease [2–5], with a population-attributable fraction of approximately 5% [6]. Targeting hypertension may be a promising strategy to delay or prevent dementia, given its high prevalence and the wide availability of antihypertensive medication (AHM) worldwide [7]. Class-specific mechanisms of AHM may contribute to a differential effect on dementia risk [8–10], potentially explaining some of the inconsistent results of previous hypertension trials and meta-analyses [11–13]. A network meta-analysis of studies comparing dementia risks between users of different AHM classes suggests that users of angiotensin receptor blockers (ARBs) and calcium channel blockers (CCBs) had a 12–17% lower risk of dementia compared with individuals using angiotensin-converting enzyme (ACE) inhibitors and beta-blockers but less so versus diuretics [14].

A potential mechanism underlying these findings is the ‘angiotensin hypothesis’, which suggests that antihypertensive agents that stimulate the angiotensin II type 2 (AT2) and 4 (AT4) receptors, including ARBs, dihydropyridine CCBs and thiazide diuretics, may reduce dementia risk by inhibiting neuronal damage and preserving memory function [15,16]. We observed that, specifically, these angiotensin II (ATII)-stimulating antihypertensive users had a 45% lower dementia risk compared with users of other AHM types in the Prevention of Dementia by Intensive Vascular care (preDIVA) population [15]. This finding was recently replicated in the SPRINT-MIND trial population, wherein ATII-stimulating AHM users had a 24% lower dementia risk when compared with other AHM users [16]. Moreover, we previously observed that individuals who used ARBs and CCBs at baseline had an approximately 40% lower dementia risk compared with individuals using other AHM types over 6.7 years of follow-up [17].

It is unclear how these associations are affected by follow-up time, and whether they are sustained over long periods. A network meta-analysis suggests that protective effects are particularly observed in studies with longer follow-up [14]. Crucially, however, these findings were nearly exclusively based on studies with a maximum follow-up of approximately 7 years. Duration of follow-up may be especially important in dementia, as it can develop insidiously over many years, implying that any protective effects of AHM classes may only become apparent in the long-term. Alternatively, protective associations may wear off over time, and/or attenuate because of changes in blood pressure and AHM regimen.

The preDIVA observational extension (POE) study yields longitudinal data on AHM use and dementia status of 3526 older adults up to 12 (median 10.4) years of follow-up. The aim of this study is to assess whether the associations between ARBs and CCBs, as well as dihydropyridine CCBs and ATII-stimulating AHM as a group and dementia persist, attenuate or increase over up to 12 years of follow-up, using the POE data.

## METHODS

For the current study, we have used data from the preDIVA study and its observational extension. The initial preDIVA cluster-randomized controlled trial assessed the effect of intensive vascular care versus standard care on the incidence of all-cause dementia after a median intervention period of 6.7 years in 3526 Dutch community-dwelling, older adults (70–78 years) without dementia [18]. In the subsequent POE study, we included former preDIVA participants who had not deceased or developed dementia during that period. After adding another four years of observational follow-up, leading to a median follow-up of 10.4 years since baseline, information on dementia status or death could be obtained in 3491 (99.0%) and 3521 (99.9%) participants, respectively. The study protocols and outcomes of the preDIVA and POE studies have been reported in more detail elsewhere [18–20]. The preDIVA trial was registered at the ISRCTN-registry (no.29711771). Both preDIVA and POE were approved by the medical ethics committee of the Academic Medical Centre, Amsterdam, the Netherlands. Participants gave written informed consent at the respective preDIVA and POE baselines. As the preDIVA trial results for dementia and mortality were similar between the intervention and control groups, for the current analysis, we considered the trial population as a single cohort, using additional adjustment for randomization group.

### Independent variables

Demographics and data on other independent variables were collected at baseline and 2, 4 and 6–8 years thereafter. Data on medication use and medical (cardiovascular) history gathered during these visits were crosschecked with participants’ electronic health records (EHR). Blood pressure (BP) was assessed by taking the mean of two baseline BP measurements, performed at the same arm in sitting position with an automated BP monitor (M6; OMRON Healthcare Co., Ltd., Kyoto, Japan) [21]. BMI and low-density lipoprotein (LDL) cholesterol were measured using standardized devices and procedures. Self-reported data on education, smoking, and physical activity were defined according to WHO criteria.

We identified five main classes of AHM: ACE inhibitors, ARBs, beta-blockers, CCBs and diuretics. We further distinguished between use of dihydropyridine and nondihydropyridine CCBs, and between ATII-stimulating and inhibiting AHM, as this was differentially associated with dementia risk in previous studies [15,16,22]. ARBs, dihydropyridine CCBs or thiazide diuretics increase Angiotensin II levels and were thus included in the ATII-stimulating group [15,23–25]. AHM were (sub)-categorized into classes according to WHO Anatomical Therapeutic Chemical (ATC) codes (supplementary Table 1) [26].

### Outcome assessment

Diagnosis of dementia was defined according to the *Diagnostic and Statistical Manual of Mental Disorders IV* (DSM-IV). An independent outcome adjudication committee, blinded for study allocation, evaluated the diagnosis of dementia and re-evaluated the diagnosis after 1 year, to minimize the risk of false-positive diagnoses. In the POE study, the municipal death registry was consulted first. Of those participants who had deceased since the final visit of preDIVA, information on the development of dementia since the end of preDIVA was obtained from the general practitioner. Those still alive were asked to participate in the telephone interview of cognitive status (TICS), which is an 11-item, validated screening tool (maximum score = 41) [27]. For participants with a TICS score greater than 30 and no known diagnosis of dementia, we assumed no dementia had occurred. For those with a TICS score 30 or less or missing score, the EHR of the general practitioner was checked for a diagnosis of dementia [28].

### Statistical analysis

We included all participants who used AHM at preDIVA baseline, with available baseline data on AHM use, covariates and outcome of dementia and mortality. Individuals who did not use AHM at baseline were excluded to limit the potential influence of selective dropout. In order to focus on the differential effects between AHM classes, we compared use of specific AHM classes with use of any other AHM classes. Participants who used multiple classes simultaneously (for instance, those using fixed combination therapy) were represented in multiple classes or subgroups at once. The association between AHM class and dementia incidence was analysed using Cox proportional hazards regression models, using number of years from baseline to diagnosis of dementia, time of death, or date of outcome assessment as timescale. Model 1 was unadjusted. In model 2, we adjusted for age, sex history of cardiovascular disease (CVD) (i.e. myocardial infarction, stroke and/or transient ischemic attack), and type 2 diabetes. In model 3, we additionally adjusted for randomization group and number of used AHM classes, as indicator for the intensity of treatment. Sensitivity- and subgroup analyses were adjusted according to model 2. In order to compare potential differences between short- and long-term results, we repeated the main analysis with a shorter follow-up period. Short-term was defined using the median follow-up of participants who developed dementia, ensuring even distribution of cases on either side of the cut-off value.

Several sensitivity analyses were performed to assess the robustness of the main analyses. First, we included all AHM classes in one model, to adjust for concurrent use of multiple AHM classes. Second, to assess the potential influence of AHM class changes during follow-up, we performed a sensitivity analysis for stable users, defined as use of the same AHM class at baseline and during at least one follow-up visit of preDIVA. Third, to assess the influence of the competing risk of death, we used the cause-specific hazard approach, repeating all analyses with mortality and dementia/mortality combined as outcomes. In a post hoc sensitivity analysis, we compared use of ARBs and/or CCBs with use of any other AHM. As both classes have a presumed negative association with dementia risk, we used this approach to limit potential concealment of the effect between use of ARBs and dementia risk by use of CCBs in the reference group, and vice versa. Finally, we included dihydropyridine CCBs and ATII-stimulating AHM in (prespecified) sensitivity- and subgroup analyses.

Subgroup analyses were performed for age (cut-off 75 years at baseline, based on the mean age at baseline in preDIVA), for participants with(out) CVD, type 2 diabetes, (un)controlled hypertension (SBP cut-off at 150 mmHg, based on the prevailing primary care guideline on hypertension at the start of the preDIVA study [29]) at baseline, and on monotherapy vs. combination therapy, as these may be proxies for different cardiovascular risk profiles, with different dementia risks. Finally, a subgroup analysis for sex was performed, as previous studies have suggested that the relation between the RAS system and development of dementia may be different between male and female individuals [23].

No imputations were deemed necessary, because of the low number of missing values in both the preDIVA trial and observational follow-up (Supplementary Table 2). All analyses were performed in RStudio(v1.3) based on R (v4.0.2; R Foundation for Statistical Computing, Vienna, Austria).

## RESULTS

In total, 1907 (54.1%) AHM users out of 3526 participants were included in the analyses. Mean age of participants at baseline was 74.5 (±2.5) years, 1027 (53.9%) were female individuals. Mean SBP was 156.2 (±21.5) mmHg. Including combination therapy, 620 (32.5%) participants used ACE inhibitors, 390 (20.5%) ARBs, 958 (50.2%) beta-blockers, 512 (26.8%) CCBs and (51.1%) 974 (51.1%) diuretics. More specifically, within the CCB group, 399 (77.9%) used dihydropyridines and 115 (22.5%) nondihydropyridines. Within the diuretic group, 752 (77.4%) used thiazides. Table 1 gives an overview of baseline data for participants in each AHM class.

**TABLE 1:** Baseline characteristics of participants with different classes of antihypertensive medication

		Total (*N* = 1907)	ACEi (*N* = 620)	ARB (*N* = 390)	Beta-blocker (*N* = 958)	CCB (*N* = 512)	Diuretic (*N* = 974)	Dihydropyridine CCB (*N* = 399)	ATII-stimulating AHM (*N* = 1180)
Sociodemographic									
Age (years)	Mean ± SD [Range]	74.5 ± 2.5 [69–80]	74.5 ± 2.5 [69–80]	74.3 ± 2.5 [69–79]	74.4 ± 2.5 [69–80]	74.5 ± 2.5 [69–80]	74.5 ± 2.5 [69–80]	74.4 ± 2.5 [69–80]	74.4 ± 2.5 [69–80]
Sex (female)	*N* (%)	1027 (53.9)	280 (45.2)	219 (56.2)	492 (51.4)	273 (53.3)	591 (60.7)	215 (53.9)	682 (57.8)
MMSE	Median [IQR]	28 [27–29]	28 [27–29]	29 [27–29]	28 [27–29]	29 [27–29]	29 [27–29]	29 [27–29]	29 [27–29]
Cardiovascular risk factors and medication use									
CVD history (yes)	N (%)	947 (49.7)	315 (50.8)	184 (47.2)	589 (61.5)	289 (56.4)	438 (45.0)	204 (51.1)	517 (43.8)
DM history (yes)	N (%)	501 (26.3)	233 (37.6)	110 (28.2)	240 (25.1)	154 (30.1)	301 (30.9)	126 (31.6)	332 (28.1)
SBP (mmHg)	Mean ± SD [Range]	156.2 ± 21.5 [100–232.5]	156.6 ± 21.9 [100–233]	156.5 ± 23.1 [103–222]	156.6 ± 22.5 [100–233]	155.8 ± 20.4 [109–218]	155.9 ± 21.5 [100–233]	157.5 ± 20.3 [109–218]	157.7 ± 21.0 [101–233]
DBP (mmHg)	Mean ± SD [Range]	81.4 ± 11.2 [50–131]	81.4 ± 11.9 [52–131]	81.5 ± 11.2 [55–118]	80.9 ± 11.4 [50–131]	79.4 ± 10.4 [52–125]	81.3 ± 10.8 [52–119]	79.7 ± 10.4 [52–125]	81.6 ± 10.9 [52–125]
BMI (kg/m^2^)	Mean ± SD	28.4 ± 4.3	28.2 ± 4.1	29.1 ± 4.6	28.3 ± 4.0	28.5 ± 4.2	28.9 ± 4.5	28.6 ± 4.1	28.7 ± 4.4
LDL (mg/dl)	Mean ± SD	112.0 ± 38.6	108.1 ± 34.8	112.0 ± 38.6	108.1 ± 34.8	108.1 ± 34.8	112.0 ± 38.6	108.1 ± 34.8	112.0 ± 38.6
Current smoking (yes)	*N* (%)	232 (12.2)	79 (12.7)	40 (10.3)	121 (12.6)	66 (12.9)	117 (12.0)	52 (13.0)	141 (11.9)
Physically active (yes)	*N* (%)	1565 (82.1)	493 (79.5)	321 (82.3)	797 (83.2)	414 (80.9)	778 (79.9)	325 (81.5)	974 (82.5)
Number of AHM	Median [IQR]	2 [2–3]	2 [2–3]	2 [2–3]	2 [1–3]	2 [2–3]	2 [2–3]	2 [2–3]	2 [2–3]

Individual participants are represented in different classes of antihypertensive medication when they use combination therapy. Data are presented as numbers (percentage), mean ± SD, median (IQR) or ranges. Physical activity was self-reported and defined according to WHO criteria. ACEi, angiotensin-converting enzyme inhibitor; AHM, antihypertensive medication; ARB, angiotensin II receptor blocker; BP, blood pressure; CCB, calcium channel blocker; CVD, cardiovascular disease; DM, diabetes mellitus; LDL, low-density lipoprotein; MMSE, Mini-Mental State Examination.

**FIGURE 1:**
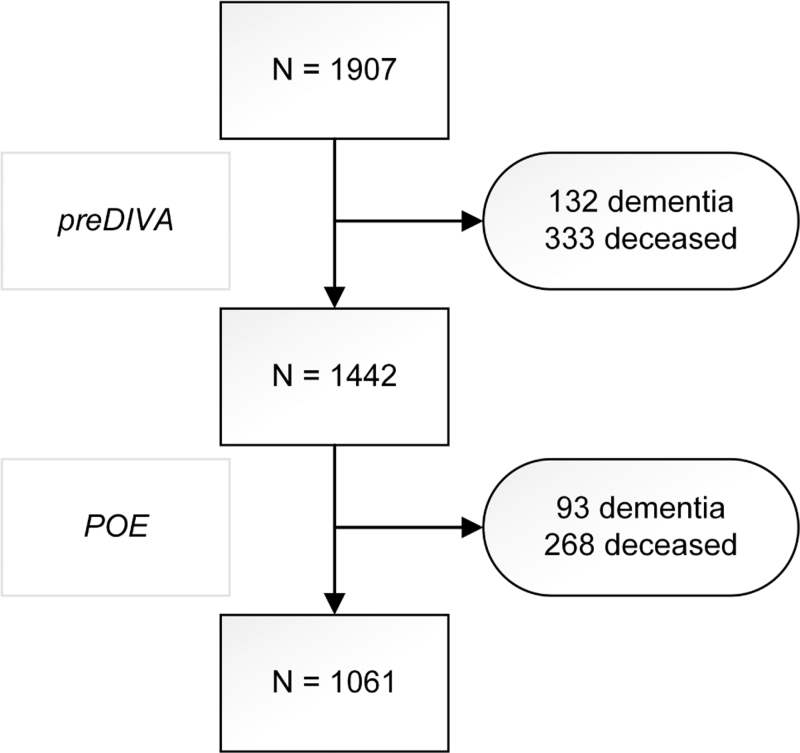
Overview of outcome assessment. Participants who had dementia and subsequently deceased, were included in the number of people with dementia only. AHM, antihypertensive medication; POE, preDIVA observational extension; preDIVA, prevention of dementia by intensive vascular care; RCT, randomized controlled trial.

Among all participants, after a median 10.4 years (range 0.2–12.8, IQR 6.8–11.0) of follow-up, 225 (11.8%) participants had developed dementia (Fig. 1). Risk of dementia was not significantly different for any of the AHM classes of interest as compared with use of any other AHM class in the crude and adjusted model (Table 2). Point estimates for use of ARBs (hazard ratio = 0.75, 95% CI = 0.53–1.07), dihydropyridine CCBs (hazard ratio = 0.73, 95% CI = 0.51–1.04) and ATII-stimulating AHM (hazard ratio = 0.80, 95% CI = 0.61–1.04) suggested a negative association with incident dementia (Table 2 and Fig. 2  , and Supplementary Figure 1).

**TABLE 2:** Association between use of a specific antihypertensive medication class and incident dementia, compared with use of any other antihypertensive medication

	Dementia cases (%) in AHM class of interest	Dementia cases (%) in other AHM users	Crude model HR (95% CI)	Model 2 HR (95% CI)
ACEi	72/620 (11.6)	153/1287 (11.9)	1.09 (0.82–1.44)	1.07 (0.81–1.43)
ARB	37/390 (9.5)	188/1517 (12.4)	0.75 (0.53–1.07)	0.75 (0.53–1.07)
Beta-blocker	113/958 (11.8)	112/949 (11.8)	1.01 (0.78–1.31)	0.99 (0.76–1.30)
CCB	58/512 (11.3)	167/1395 (12.0)	0.96 (0.71–1.29)	0.92 (0.68–1.25)
Diuretic	117/974 (12.0)	108/933 (11.6)	1.07 (0.82–1.39)	1.03 (0.79–1.34)
Dihydropyridine CCB	37/399 (9.3)	188/1508 (12.5)	0.74 (0.52–1.05)	0.73 (0.51–1.04)
ATII-stimulating AHM	129/1180 (10.9)	96/727 (13.2)	0.81 (0.62–1.05)	0.80 (0.61–1.04)

Median follow-up: 10.4 years. Model 2: adjusted for age, sex, history of cardiovascular disease, and history of diabetes mellitus. The dementia cases (percentages) represent the number of participants with incident dementia from the participants using the AHM class of interest. ATII-stimulating AHM include ARBs, dihydropyridine CCBs and thiazide diuretics. ACEi, angiotensin-converting enzyme inhibitor; AHM, antihypertensive medication; ARB, angiotensin II receptor blocker; ATII, angiotensin II; CCB, calcium channel blocker; CI, confidence interval; HR, hazard ratio.

**FIGURE 2:**
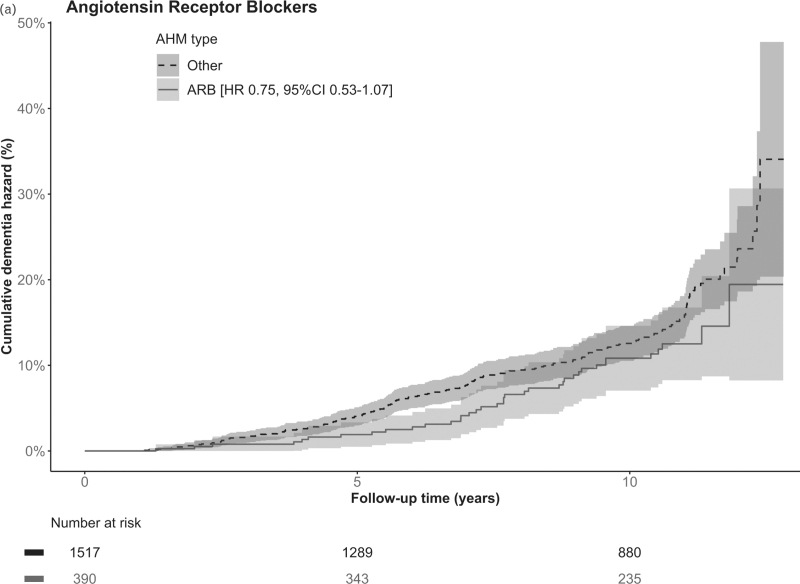
Cumulative hazard of dementia for angiotensin receptor blockers, dihydropyridine calcium channel blockers and angiotensin II-stimulating antihypertensive medication. (a) ARBs, (b) dihydropyridine CCBs and (c) ATII-stimulating AHM versus any other AHM classes. AHM, antihypertensive medication; ARB, angiotensin receptor blocker; ATII, angiotensin II; CCB, calcium channel blocker; CI, confidence interval; HR, hazard ratio.

**FIGURE 2 (Continued):**
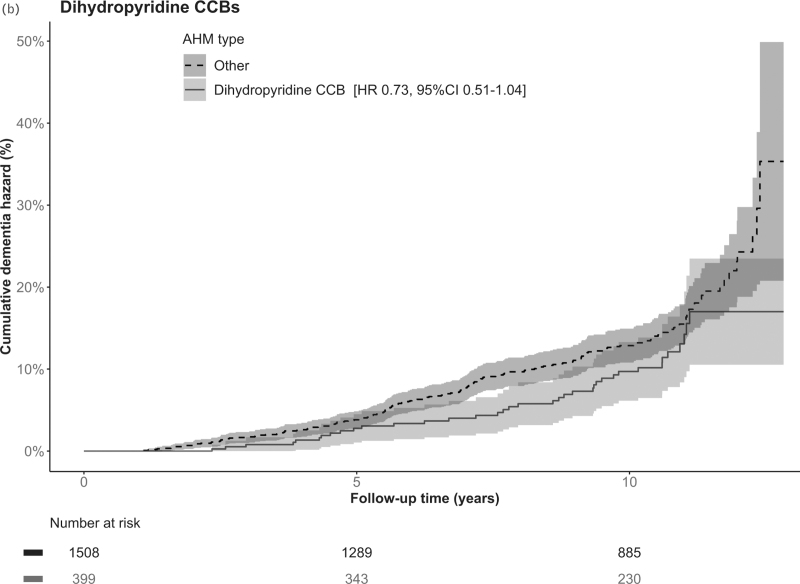
Cumulative hazard of dementia for angiotensin receptor blockers, dihydropyridine calcium channel blockers and angiotensin II-stimulating antihypertensive medication. (a) ARBs, (b) dihydropyridine CCBs and (c) ATII-stimulating AHM versus any other AHM classes. AHM, antihypertensive medication; ARB, angiotensin receptor blocker; ATII, angiotensin II; CCB, calcium channel blocker; CI, confidence interval; HR, hazard ratio.

**FIGURE 2 (Continued):**
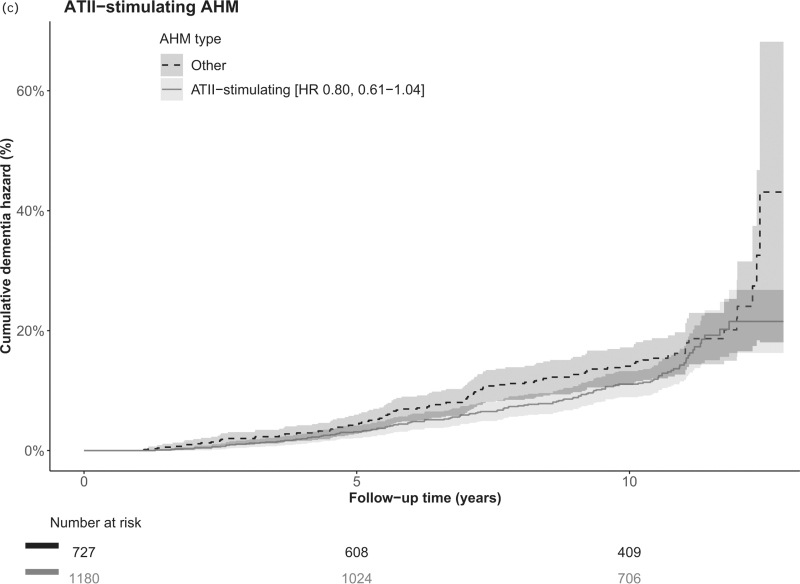
Cumulative hazard of dementia for angiotensin receptor blockers, dihydropyridine calcium channel blockers and angiotensin II-stimulating antihypertensive medication. (a) ARBs, (b) dihydropyridine CCBs and (c) ATII-stimulating AHM versus any other AHM classes. AHM, antihypertensive medication; ARB, angiotensin receptor blocker; ATII, angiotensin II; CCB, calcium channel blocker; CI, confidence interval; HR, hazard ratio.

Short-term, with follow-up cut-off at 7 years (median follow-up of dementia cases), use of ARBs (hazard ratio = 0.54, 95% CI = 0.31–0.94), CCBs (hazard ratio = 0.60, 95% CI = 0.37–0.97), dihydropyridine CCBs (hazard ratio = 0.52, 95% CI = 0.30–0.91) and ATII-stimulating AHM (hazard ratio = 0.68, 95% CI = 0.47–1.00) was associated with reduced dementia risk (Supplementary Table 4). Results from the main analyses remained largely unchanged after additional adjustment for number of AHM and randomisation group (Supplementary Table 5) and when mutually adjusting for all main AHM classes in one model (Supplementary Table 6). When restricting analyses to participants in the stable-use group (Supplementary Table 7), use of ATII-stimulating AHM was associated with lower dementia incidence (hazard ratio = 0.73, 95% CI = 0.52–0.99). Use of ARBs, dihydropyridine CCBs and ATII-stimulating AHM were not associated with increased mortality rates (hazard ratio = 0.94, 95% CI = 0.77–1.14; hazard ratio = 0.99, 95% CI = 0.82–1.20; hazard ratio = 0.94, 95% CI = 0.81–1.11 respectively), suggesting no evident influence of competing risk of death (Supplementary Table 8). Finally, use of ARBs and CCBs combined was associated with a lower dementia incidence (hazard ratio = 0.69, 95% CI = 0.52–0.92, Supplementary Table 9).

Associations between AHM classes and dementia were largely similar across the predefined subgroups (Supplementary Table 10), although in ARB users, the association with dementia was stronger in participants aged 75 and over (hazard ratio = 0.60, 95% CI = 0.36–0.99) when compared with those under 75 years of age at baseline (hazard ratio = 0.95, 95% CI = 0.57–1.58). In participants using ATII-stimulating AHM, the association was stronger in those with a history of diabetes (hazard ratio = 0.58, 95% CI = 0.36–0.94) when compared with individuals without history of diabetes (hazard ratio = 0.90, 95% CI = 0.65–1.25).

## DISCUSSION

### Main findings

In our population of 1907 AHM-using Dutch older adults, use of ARBs, dihydropyridine CCBs and ATII-stimulating AHM was nonsignificantly associated with 20–27% lower risk of incident dementia over a median follow-up duration of 10.4 years, and significantly with 32–48% lower risk of dementia after 7 years follow-up, when compared with use of any other AHM class.

### Interpretation of findings

The nonsignificant 20–27% lower dementia risks after median 10.4 years had decreased compared with the 30–45% lower risks over 7 years. This suggests that the associations of ARBs, (dihydropyridine) CCBs or ATII-stimulating AHM with decreased dementia risk might attenuate over time. Possibly, as many of the known risk factors for dementia are age-dependent [8,30,31], differential effects of AHM classes partly decrease with aging. Analyses stratified by age, however, do not support this hypothesis. Another explanation may be that, with increasing follow-up time, baseline data on medication use have become less reliable indicators of actual medication use. Nevertheless, sensitivity analyses in participants who used the same AHM class at baseline and during at least one follow-up visit did not substantially alter our results. Thirdly, differential effects of AHM classes on dementia risk could have a temporary nature, regardless of age. Finally, regression to the mean could in part explain the difference between associations on the short and longer term.

Baseline blood pressure levels and number of prescribed AHM were comparable between the different AHM classes users. Any differential effects between AHM classes and incident dementia we observed are, therefore, likely caused by class-specific mechanisms rather than their effect on blood pressure. Several hypotheses exist around the potential neuroprotective effects of CCBs and ARBs, ranging from their abilities to improve cerebral blood flow, reduce cerebral oxidative stress markers, and protect against neuronal death [32]. In addition, dihydropyridine CCBs and ARBs stimulate AT2 and AT4 receptors through the ATII pathway, which potentially protects against ischemia and preserve memory, respectively [15,16,33–36].

An important potential challenge in studies with dementia as outcome is the competing risk of mortality before the development of dementia. In our study, we observed associations between use of ACE inhibitors and mortality (hazard ratio1.19, 95% CI = 1.01–1.40) and dementia/mortality combined (hazard ratio 1.18, 95% CI = 1.02–1.36). This may be related to the high number of individuals with diabetes in this group. As no association between use of any other AHM class and mortality were observed, with hazard ratios around 1.0, our results appear unaffected by the competing risk of death.

### Strengths and limitations

Main strengths are the judicious assessment of the most clinically relevant outcome of incident dementia, the long follow-up period of up to 12 years, and completeness of follow-up on all-cause dementia (99.0%) and mortality (99.9%). Furthermore, our study population consists of a broadly representative sample of community-dwelling Dutch older adults [18].

A limitation is potential confounding by indication, as former Dutch guidelines recommended a stepped approach for AHM prescriptions in which ARBs and CCBs represented second or later steps in treatment. In our study, baseline blood pressure values were comparable across classes, but beta-blockers, ACE inhibitors, and ARBs were more often prescribed among specific groups, including those with a history of CVD or diabetes. To address this issue, we adjusted for CVD and diabetes history in the main model, which did not change the results of the crude analyses. Additional adjustment for number of AHM classes did not change the results. Also, results were highly comparable in subgroup analyses for participants with and without diabetes, a history of CVD and uncontrolled hypertension.

A second limitation is the lack of complete data on medication history prior to baseline assessment, medication adherence and dosage. In the main analysis, we only used data on AHM use collected at baseline, ignoring intermediate changes in AHM use. We repeated the main analysis in a sample of participants who used the same AHM class at baseline and at least one follow-up visit and observed similar results. The available data did not allow for a more thorough analysis on the effects of postbaseline AHM class switching and medication exposure over time.

### Comparison with previous studies

The hazard ratios for incident dementia ranging between 0.73 and 0.80 we found, are in line with findings from previous studies on class-specific effects of AHM. Two individual participant data (IPD) studies with dementia as secondary outcome compared use of various AHM classes with use of any other AHM class. Both studies reported negative, albeit nonsignificant associations with incident dementia. One study found that use of ARBs was associated with a 12–24% lower dementia risk and the other reported 7–24% lower ORs for ARBs and CCBs [37,38]. A recent network meta-analysis compared use of various AHM classes to each other and demonstrated that use of CCBs and ARBs was associated with a 12–17% reduced dementia risk compared with ACE inhibitors and beta blockers, but less so versus diuretics (7–11%). However, all but one included studies had a follow-up period of less than approximately 7 years and most applied nonuse of AHM classes, including individuals who did not use any AHM at all, as reference groups, hindering accurate comparison with our results [14,22,39,40]. One study with a follow-up of over 10 years compared use of CCBs with use of other AHM classes and found a significant 19% reduction of dementia risk in those using CCBs [41]. Our study is the first to assess the sustainability of class-specific associations between various AHM classes and incident dementia over a prolonged period of time.

In conclusion, in our study population of Dutch community-dwelling older persons, we did not observe statistically significant associations between use of any specific AHM class and dementia risk over a median 10.4 years of follow-up, although point estimates for ARBs, dihydropyridine CCBs and ATII-stimulating AHM suggest a lower risk of dementia when compared with use of any other AHM class. Possibly, significant associations observed in the short-term represented effects that were to some extent temporary, or could not be replicated over the complete follow-up period because baseline AHM data were not fully representative of actual medication use over time. However, even temporary effects, resulting in delayed manifestation of dementia, could be meaningful to both individuals and society. Further studies assessing the sustainability of class-specific associations in older adults should constitute detailed registration of AHM use over time, to account for intermediate class-changes and to assess potential dose–effect relationships.

## ACKNOWLEDGEMENTS

We would like to thank all participants of the preDIVA study, all practice nurses who delivered the intervention, and all general practitioners involved in the care for the participants. We thank Ursula Kröder, Marije Voermans and Hanneke Muiser for the extended follow-up assessments.

Financial disclosure: The preDIVA Trial was supported by the Dutch Ministry of Health, Welfare and Sports (grant number 50-50110-98-020), the Dutch Innovation Fund of Collaborative Health Insurances (grant number 05-234) and Netherlands Organization for Health Research and Development (grant number 62000015).

E.R.is funded by a personal grant from The Netherlands Organization for Health Research and Development (grant number 91718303).

The preDIVA observational extension was supported by Alzheimer Nederland, project number wE.09-2017-08.

J.S. was supported by the Netherlands Organisation for Health Research and Development (grant number 839110025).

E.E. was supported by a grant from the Amsterdam University Medical Center Research Council (grant number 180207).

The sponsors had no role in the design and conduct of the study; in the collection, analysis and interpretation of data; in the preparation of the manuscript; or in the review or approval of the manuscript.

### Conflicts of interest

There are no conflicts of interest.

## Supplementary Material

Supplemental Digital Content
