# Supplementary material for: Antihypertensive medication classes and the risk of dementia over a decade of follow-up
Source: J Hypertens. 2022 Nov 18;41(2):262–70. doi: 10.1097/HJH.0000000000003324 (PMC9799049; doi:10.1097/HJH.0000000000003324)
Supplement: Supplemental Digital Content [file jhype-41-262-s001.docx]

**Supplementary table 1.** AHM classes and corresponding ATC codes.

| **AHM class** | **ATC codes** |
| --- | --- |
| ACEi | C09A; C09B |
| ARB | C09C; C09D |
| Beta-blocker | C07A; C07B; C07C; C07D; C07E; C07F; C09BX02, C09BX04; C09DX06 |
| CCB | C08C, C08D, C08E, C08G; C07FB; C09XA53; C09XA54; C09DX01; C09DX03; C09DX07; C09DB; C09BX04; C09BX01; C09BX03; C09BB |
| Diuretic | C03A, C03B, C03C, C03D, C03E, C03X; C02L; C07B; C07C; C07D; C08G; C09BA; C09BX01; C09BX03; C09DA; C09DX01; C09DX03; C09DX07; C09XA52; C09XA54 |
| Other | C02A, C02B, C02C, C02D, C02K, C02L, C02N, C09X |
| Dihydropyridine  CCB | C08G; C07FB; C09XA53; C09XA54; C09DX01; C09DX03; C09DX06; C09DX07; C09DB; C09BX01; C09BX03; C09BX04; C09BB02; C09BB03; C09BB04; C09BB06; C09BB07; C09BB12 |
| Angiotensin II-  stimulating AHM | C02L; C03A; C03EA01; C03EA02; C03EA03; C03EA04; C03EA05;C03EA07; C03EA013; C03EA014; C07B; C07D; C07FB; C08CA; C08G; C09BA; C09BB02; C09BB03; C09BB04; C09BB05; C09BB06; C09BB07; 09BB12; C09BX01; C09BX03; C09BX03; C09BX04; C09CA; C09DA; C09DB; C09DX; C09XA52; C09XA53; C09XA54 |

Angiotensin II-stimulating AHM include ARBs, dihydropyridine CCBs and thiazide diuretics.

AHM = antihypertensive medication; ATC = Anatomical Therapeutic Chemical; ACEi = angiotensin-converting enzyme inhibitor; ARB=angiotensin II receptor blocker; CCB = calcium channel blocker.

**Supplementary table 2.** Number of participants with missing data for each variable of interest.

| Variables | Participants with missing data (%) |
| --- | --- |
| Age | 0 (0) |
| Dementia | 21 (1.1) |
| History of CVD | 24 (1.2) |
| Diabetes mellitus | 0 (0) |
| Systolic blood pressure | 0 |
| Diastolic blood pressure | 0 |
| Smoking | 3 (0.2) |
| Physical activity | 41 (2.1) |
| LDL | 53 (2.7) |
| BMI | 1 (0.1) |
| MMSE | 3 (0.2) |

Data presented for all participants who used who used antihypertensive medication at baseline (n=1953).

CVD = cardiovascular disease; LDL = low-density lipoprotein; BMI = Body-Mass index; MMSE = Mini-Mental State Examination.

**Supplementary table 3.** Number of antihypertensive medication (AHM) classes and combinations of AHM at baseline.

|  | | **ACEi (N=620)** | **ARB (N=390)** | **Beta-blocker (N=958)** | **CCB (N=512)** | **Diuretic (N=974)** |
| --- | --- | --- | --- | --- | --- | --- |
| **Number of AHM classes,**  **N (%)** | |  |  |  |  |  |
| 1 | 137 (22.1) | | 90 (23.1) | 282 (29.4) | 104 (20.3) | 182 (18.7) |
| 2 | 247 (39.8) | | 164 (42.1) | 364 (38.0) | 172 (33.6) | 457 (46.9) |
| 3 | 181 (29.2) | | 101 (25.9) | 233 (24.3) | 157 (30.7) | 255 (26.2) |
| ≥4 | 55 (8.9) | | 35 (9.0) | 79 (8.2) | 79 (15.4) | 80 (8.2) |
| **AHM classes** |  | |  |  |  |  |
| ACEi | 620 (100.0) | | 13 (3.3) | 258 (26.9) | 161 (31.4) | 342 (35.1) |
| ARB | 13 (2.1) | | 390 (100.0) | 150 (15.7) | 107 (20.9) | 201 (20.6) |
| Beta-blocker | 258 (41.6) | | 150 (38.5) | 958 (100.0) | 225 (43.9) | 434 (44.6) |
| CCB | 161 (26.0) | | 107 (27.4) | 225 (23.5) | 512 (100.0) | 230 (23.6) |
| Diuretic | 342 (55.2) | | 201 (51.6) | 434 (45.3) | 230 (44.9) | 974 (100.0) |
| Cholesterol lowering medication | 346 (55.8) | | 183 (46.9) | 528 (55.1) | 266 (52.0) | 448 (46.0) |

Median follow-up: 10.4 years. Individual participants are represented in different classes of antihypertensive medication when they use combination therapy.

ACEi = angiotensin-converting enzyme inhibitor; AHM = antihypertensive medication; ARB = angiotensin II receptor blocker; CCB = calcium channel blocker.

**Supplementary figure 1.** Cumulative hazard of dementia for ACE inhibitors, beta-blockers, calcium channel blockers and diuretics

**
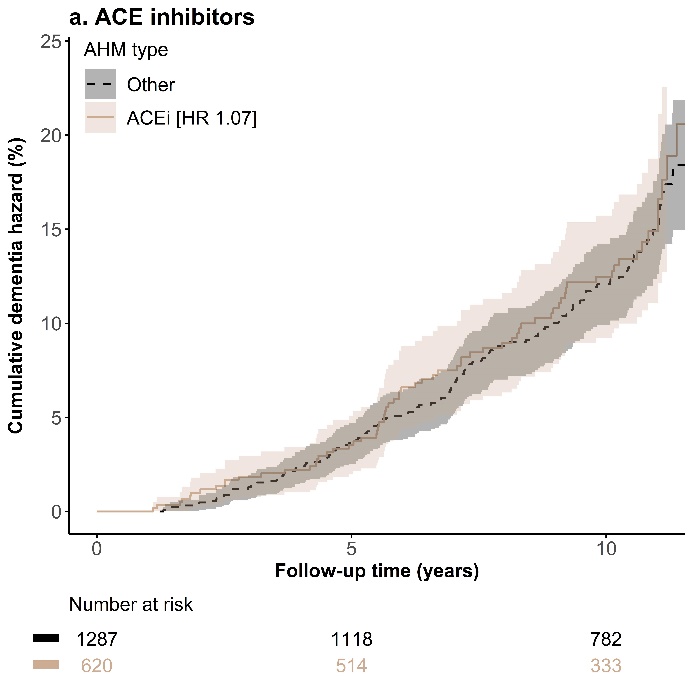
**
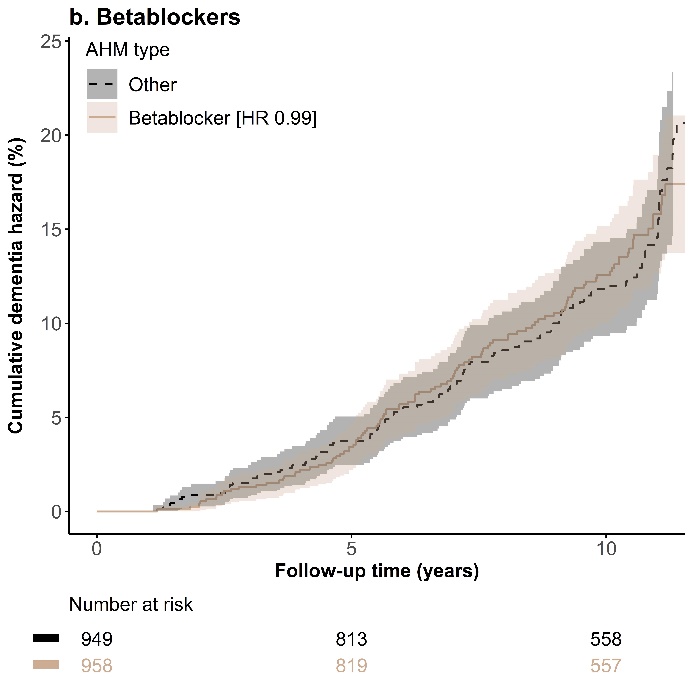


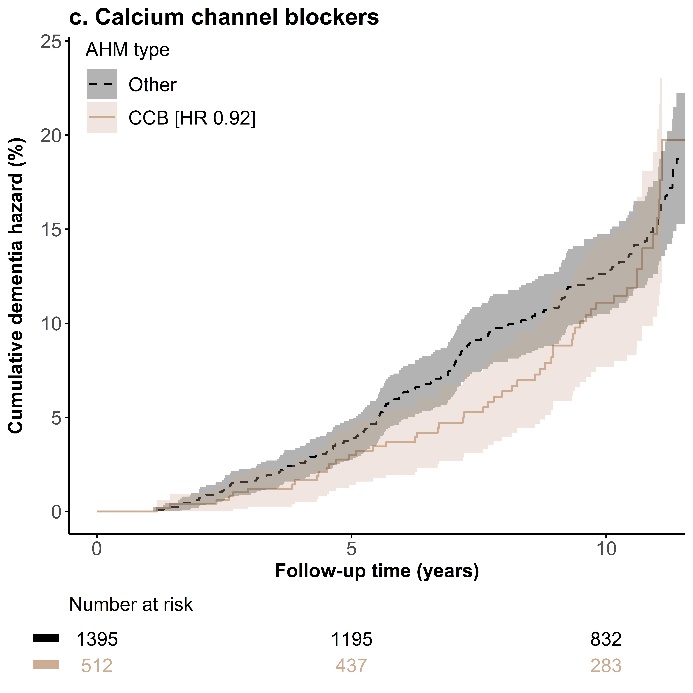
**
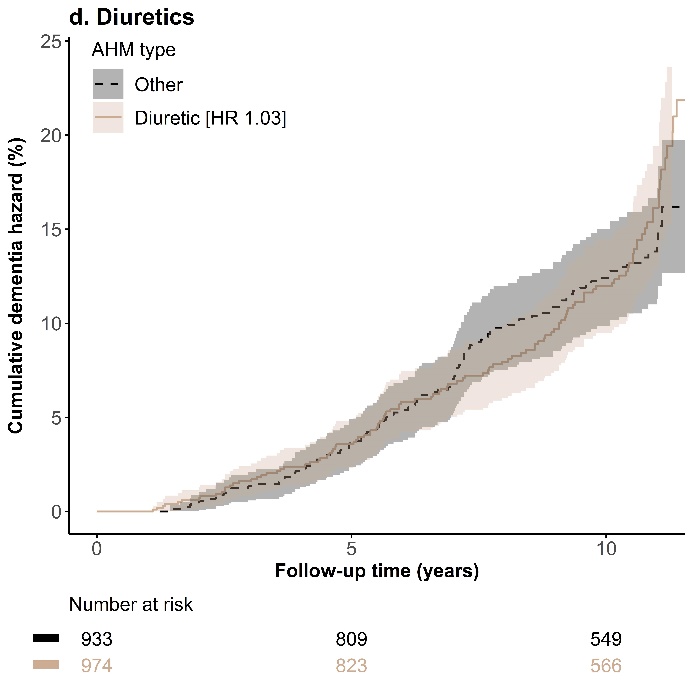
**

a. ACEi, b. beta-blockers, c. CCBs, d. diuretics (brown) versus any other AHM classes (grey).

ACEi = angiotensin-converting enzyme inhibitor, AHM = antihypertensive medication, HR = hazard ratio;CCB = calcium channel blocker, AHM = antihypertensive medication, HR = hazard ratio.

**Supplementary table 4.** Short-term associations between use of a specific antihypertensive medication class and incident dementia, compared with use of any other antihypertensive medication classes.

|  | Dementia cases (%) | HR (95% CI) |
| --- | --- | --- |
| ACEi | 39/620 (6.3) | 1.12 (0.75-1.66) |
| ARB | 14/390 (3.6) | 0.54 (0.31-0.94) |
| Beta-blocker | 58/958 (6.4) | 1.05 (0.72-1.54) |
| CCB | 21/512 (4.1) | 0.60(0.37-0.97) |
| Diuretic | 57/974 (5.9) | 0.96 (0.66-1.40) |
|  |  |  |
| Dihydropyridine CCB | 18/399 (3.5) | 0.52 (0.30-0.91) |
| AT II-stimulating AHM | 36/1180 (3.1) | 0.68 (0.47-1.00) |

Follow-up cut off at 7 years. Model 2: adjusted for age, sex, history of cardiovascular disease, and history of diabetes mellitus. The dementia cases (percentages) represent the number of participants with incident dementia from the participants using the AHM class of interest. ATII-stimulating AHM include ARB’s, dihydropyridine CCB’s and thiazide diuretics.

ACEi = angiotensin converting enzyme inhibitor; AHM = antihypertensive medication; ARB = angiotensin II receptor blocker; AT II = angiotensin II; CCB = calcium channel blocker; CI = confidence interval; HR = hazard ratio.

**Supplementary table 5.** Associations between use of antihypertensive medication (AHM) classes
and incident dementia, compared with use of any other AHM class – maximally adjusted model.

|  | Dementia cases (%) in AHM class of interest | Dementia cases (%) in other AHM users | Model 3  HR (95% CI) |
| --- | --- | --- | --- |
| ACEi | 72/620 (11.6) | 153/1287 (11.9) | 1.13 (0.83-1.52) |
| ARB | 37/390 (9.5) | 188/1517 (12.4) | 0.76 (0.53-1.09) |
| Beta-blocker | 113/958 (11.8) | 112/949 (11.8) | 1.05 (0.78-1.37) |
| CCB | 58/512 (11.3) | 167/1395 (12.0) | 0.96 (0.69-1.32) |
| Diuretic | 117/974 (12.0) | 108/933 (11.6) | 1.10 (0.81-1.50) |
|  |  |  |  |
| Dihydropyridine CCB | 37/399 (9.3) | 188/1508 (12.5) | 0.73 (0.50-1.07) |
| ATII-stimulating AHM | 129/1180 (10.9) | 96/727 (13.2) | 0.79 (0.58-1.08) |

Median follow-up: 10.4 years. Model 3: adjusted for age, sex, history of cardiovascular disease, history of diabetes mellitus, number of antihypertensive drugs, and randomization group. ATII-stimulating antihypertensives include ARBs, dihydropyridine CCBs, and thiazide diuretics.

ACEi = angiotensin converting enzyme inhibitor; AHM = antihypertensive medication; ARB = angiotensin II receptor blocker; ATII = angiotensin II; CCB = calcium channel blocker; CI = confidence interval; HR = hazard ratio.

**Supplementary table 6.** Associations between use of

antihypertensive medication class and dementia,

mutually adjusted for use of multiple classes.

|  | HR (95% CI) |  |
| --- | --- | --- |
| ACEi | 0.98 (0.72-1.34) |  |
| ARB | 0.74 (0.51-1.08) |  |
| Beta-blocker | 0.96 (0.73-1.27) |  |
| CCB | 0.92 (0.68-1.24) |  |
| Diuretic | 1.01 (0.77-1.33) |  |

Cox proportional hazard model adjusted for age, sex, history of cardiovascular disease, and history of diabetes mellitus.

ACEi = angiotensin converting enzyme inhibitor; ARB = angiotensin receptor blocker; CCB = calcium channel blocker; CI = confidence interval; HR = hazard ratio.

**Supplementary table 7.** Associations between use of antihypertensive medication classes and

dementia within stable users.

|  | Stable- /total users (%) | Dementia cases (%) | HR (95% CI) |
| --- | --- | --- | --- |
| ACEi | 418/620 (67.4) | 50/418 (12.0) | 1.12 (0.80-1.57) |
| ARB | 302/390 (77.4) | 29/302 (9.6) | 0.78 (0.52-1.16) |
| Beta-blocker | 713/958 (74.4) | 87/713 (12.2) | 1.01 (0.74-1.38) |
| CCB | 372/512 (72.7) | 40/372 (10.7) | 0.87 (0.61-1.24) |
| Diuretic | 685/974 (70.3) | 73/685 (10.6) | 0.79 (0.58-1.08) |
|  |  |  |  |
| Dihydropyridine CCB^a^ | 286/399 (71.7) | 27/286 (9.4) | 0.77 (0.51-1.17) |
| ATII-stimulating AHM^a^ | 883/1180 (74.8) | 91/883 (10.3) | 0.73 (0.52-0.99) |

Median follow-up: 10.4 years. Cox proportional hazard model adjusted for age, sex, history of cardiovascular disease, and history of diabetes mellitus. Stable users as defined as using the same antihypertensive medication group at baseline and at one or more preDIVA follow-up visits. ATII-stimulating AHM include ARBs, dihydropyridine CCBs and thiazide diuretics.

ACEi = angiotensin converting enzyme inhibitor; ARB = angiotensin II receptor blocker; CCB = calcium channel blocker; CI = confidence interval; HR = hazard ratio.

^a^Post hoc analysis.

**Supplementary table 8.** Associations between use of antihypertensive medication classes and mortality,
and dementia and mortality combined.

|  | Death cases (%) | HR death  (95% CI) | Dementia or death cases (%) | HR dementia or death (95% CI) |
| --- | --- | --- | --- | --- |
| ACEi | 237/620 (38.2) | 1.19 (1.01-1.40) | 294/620 (47.4) | 1.18 (1.02-1.36) |
| ARB | 122/390 (31.3) | 0.94 (0.77-1.14) | 154/390 (39.5) | 0.91 (0.77-1.09) |
| Beta-blocker | 323/958 (33.7) | 0.89 (0.76-1.04) | 410/958 (42.8) | 0.92 (0.80-1.05) |
| CCB | 194/512 (37.9) | 1.13 (0.95-1.34) | 237/512 (46.3) | 1.06 (0.91-1.24) |
| Diuretic | 333/974 (34.2) | 1.15 (0.98-1.34) | 424/974 (43.5) | 1.13 (0.98-1.30) |
|  |  |  |  |  |
| Dihydropyridine CCB^a^ | 135/399 (33.8) | 0.99 (0.82-1.20) | 165/399 (41.4) | 0.93 (0.79-1.11) |
| ATII-stimulating AHM^a^ | 382/1180 (32.4) | 0.94 (0.81-1.11) | 486/1180 (41.2) | 0.93 (0.81-1.07) |

Median follow-up: 10.4 years. Cox proportional hazard model adjusted for age, sex, history of cardiovascular disease, and history of

diabetes mellitus. ATII-stimulating AHM include ARBs, dihydropyridine CCBs and thiazide diuretics.

ACEi = angiotensin converting enzyme inhibitor; ARB = angiotensin receptor blocker; CCB = calcium channel blocker; CI = confidence interval; HR = hazard ratio.

^a^Post hoc analysis.

**Supplementary table 9.** Association between use of ARBs or dihydropyridine CCBs and incident dementia**.**

|  | Dementia cases (%) in AHM class of interest | Dementia cases (%) in other AHM users | Crude model  HR (95% CI) | Model 2  HR (95%CI) | Model 3  HR (95%CI) |
| --- | --- | --- | --- | --- | --- |
| ARB or dihydropyridine CCB | 692/1907 (36.3) | 65/692 (9.8) | 0.69 (0.52-0.92) | 0.69 (0.52-0.92) | 0.67 (0.49-0.92) |

Model 2: adjusted for age, sex, history of cardiovascular disease, and history of diabetes mellitus. Model 3: adjusted for age, sex, history of cardiovascular disease, history of diabetes, number of used AHM-classes, and randomization group.

AHM = antihypertensive medication; ARB = angiotensin II receptor blocker; CCB = calcium channel blocker; CI = confidence
interval; HR = hazard ratio.

**Supplementary table 10.** Subgroup analyses for the association between use of different antihypertensive medication classes and incident dementia.

|  | **Sex** | | **CVD history** | | **DM history** | | **Hypertension^b^** | | **Mono vs. multi-therapy** | | **Age** | |
| --- | --- | --- | --- | --- | --- | --- | --- | --- | --- | --- | --- | --- |
|  | **Female** | **Male** | **Yes** | **No** | **Yes** | **No** | **Uncontrolled** | **Controlled** | **Mono** | **Multi** | **< 75** | **≥ 75** |
| **ACEi**  Dementia cases (%)  HR  (95% CI) | 33/340 (9.7)  1.14  (0.72-1.79) | 39/280 (13.9)  1.04  (0.72-1.51) | 34/315 (10.8)  0.95  (0.63-1.42) | 38/305 (12.5)  1.25  (0.83-1.87) | 33/233 (14.2)  1.26  (0.77-2.04) | 39/387 (10.1)  0.99  (0.69-1.42) | 40/385 (10.4)  0.99  (0.67-1.45) | 32/235 (13.6)  1.25  (0.81-1.91) | 19/137 (13.9)  1.29  (0.77-2.16) | 53/483 (11.0)  1.01  (0.71-1.45) | 32/309 (10.4)  1.25  (0.81-1.94) | 40/311 (12.9)  0.95  (0.65-1.39) |
| P for interaction | *0.85* | | *0.36* | | *0.49* | | *0.29* | | *0.37* | | *0.38* | |
| **ARB**  Dementia cases (%)  HR  (95% CI) | 10/171 (5.8)  0.58  (0.30-1.13) | 27/219 (12.3)  0.83  (0.55-1.26) | 18/184 (9.8)  0.77  (0.46-1.27) | 19/206 (9.2)  0.71  (0.43-1.16) | 12/110 (10.9)  0.71  (0.38-1.34) | 25/280 (8.9)  0.77  (0.50-1.18) | 17/226 (7.5)  0.62  (0.37-1.04) | 20/164 (12.2)  0.93  (0.57-1.53) | 7/90 (7.8)  0.60  (0.28-1.30) | 30/300 (10.0)  0.81  (0.54-1.22) | 19/201 (9.5)  0.95  (0.57-1.58) | 18/189 (9.5)  0.60  (0.36-0.99*) |
| P for interaction | *0.42* | | *0.70* | | *0.86* | | *0.27* | | *0.52* | | *0.82* | |
| **Beta-blocker**  Dementia cases (%)  HR  (95% CI) | 45/466 (9.7)  1.08  (0.69-1.70) | 68/492 (13.8)  0.96  (0.68-1.34) | 71/589 (12.1)  1.09  (0.74-1.60) | 42/369 (11.4)  0.91  (0.62-1.34) | 33/240 (13.8)  0.90  (0.56-1.47) | 80/718 (11.1)  1.03  (0.75-1.43) | 66/576 (11.5)  1.11  (0.78-1.57) | 47/382 (12.3)  0.85  (0.56-1.30) | 33/282 (11.7)  0.97  (0.63-1.51) | 80/676 (11.8)  1.06  (0.73-1.53) | 49/493 (9.9)  1.04  (0.68-1.59) | 64/465 (13.8)  0.97  (0.68-1.37) |
| P for interaction | *0.91* | | *0.54* | | *0.80* | | *0.56* | | *0.66* | | *0.58* | |
| **CCB**  Dementia cases (%)  HR  (95% CI) | 22/239 (9.2)  0.98  (0.60-1.59) | 36/273 (13.2)  0.89  (0.61-1.30) | 34/289 (11.8)  1.00  (0.66-1.49) | 24/223 (10.8)  0.84  (0.53-1.32) | 22/154 (14.3)  1.09  (0.61-1.97) | 36/358 (10.1)  0.88  (0.59-1.31) | 28/294 (9.5)  0.80  (0.52-1.22) | 30/218 (13.8)  1.07  (0.70-1.66) | 15/104 (14.4)  1.22  (0.70-2.12) | 43/408 (10.5)  0.84  (0.58-1.22) | 20/250 (8.0)  0.79  (0.48-1.29) | 38/262 (14.5)  1.01  (0.69-1.48) |
| P for interaction | *0.89* | | *0.58* | | *0.50* | | *0.24* | | *0.30* | | *0.51* | |
| **Diuretic**  Dementia cases (%)  HR  (95% CI) | 39/383 (10.2)  1.25  (0.80-1.94) | 78/591 (13.2)  0.92  (0.66-1.29) | 50/438 (11.4)  0.90  (0.61-1.31) | 67/536 (12.5)  1.15  (0.79-1.69) | 40/301 (13.3)  0.86  (0.53-1.39) | 77/673 (11.4)  1.10  (0.80-1.53) | 72/583 (12.3)  1.25  (0.87-1.78) | 45/391 (11.5)  0.80  (0.53-1.21) | 23/182 (12.6)  0.96  (0.59-1.56) | 94/792 (11.9)  1.18  (0.79-1.78) | 45/471 (9.6)  0.95  (0.62-1.45) | 72/503 (14.3)  1.07  (0.75-1.51) |
| P for interaction | *0.28* | | *0.62* | | *0.42* | | *0.15* | | *0.66* | | *0.36* | |
| **Dihydropyridine CCB^a^**  Dementia cases (%)  HR  (95% CI) | 15/184 (8.2)  0.83  (0.47-1.45) | 22/215 (10.2)  0.69  (0.44-1.08) | 19/204 (9.3)  0.78  (0.47-1.28) | 18/195 (9.2)  0.67  (0.41-1.12) | 15/126 (11.9)  0.80  (0.45-1.43) | 22/273 (8.1)  0.67  (0.43-1.05) | 19/240 (7.9)  0.66  (0.41-1.08) | 18/159 (11.3)  0.81  (0.48-1.35) | 9/6 (13.6)  1.10  (0.55-2.20) | 28/333 (8.4)  0.64  (0.42-0.98) | 14/203 (6.9)  0.67  (0.38-1.18) | 23/196 (11.7)  0.78  (0.49-1.22) |
| P for interaction | *0.64* | | *0.66* | | *0.57* | | *0.51* | | *0.19* | | *0.36* | |
| **ATII-stimulating AHM^a^**  Dementia cases (%)  HR  (95% CI) | 43/498 (8.6)  0.80  (0.51-1.23) | 86/682 (12.6)  0.81  (0.57-1.14) | 52/517 (10.1)  0.69  (0.48-1.01) | 77/663 (11.6)  0.90  (0.60-1.34) | 39/332 (11.7)  0.58  (0.36-0.94) | 90/848 (10.6)  0.90  (0.65-1.25) | 77/739 (10.4)  0.79  (0.55-1.13) | 52/441 (11.8)  0.83  (0.55-1.27) | 36/300 (12.0)  0.91  (0.59-1.39) | 93/880 (10.6)  0.67  (0.44-1.01) | 52/591 (8.8)  0.78  (0.51-1.19) | 77/598 (13.1)  0.79  (0.55-1.12) |
| P for interaction | *0.89* | | *0.49* | | *0.13* | | *0.91* | | *0.27* | | *0.32* | |

Adjusted for age, sex, history of cardiovascular disease, and history of diabetes mellitus. ATII-stimulating AHM include ARBs, dihydropyridine CCBs and thiazide diuretics.

ACEi = angiotensin converting enzyme inhibitor; AHM = antihypertensive medication; ARB = angiotensin II receptor blocker; ATII = angiotensin II; CCB = calcium channel blocker; CI =confidence interval; CVD = cardiovascular disease; DM = diabetes mellitus; HR = hazard ratio. ^a^Post hoc analysis. ^b^Cut-off for controlled hypertension is 150 mmHg.
